# Mitochondria-derived ROS activate AMP-activated protein kinase (AMPK) indirectly

**DOI:** 10.1074/jbc.RA118.002579

**Published:** 2018-09-19

**Authors:** Elizabeth C. Hinchy, Anja V. Gruszczyk, Robin Willows, Naveenan Navaratnam, Andrew R. Hall, Georgina Bates, Thomas P. Bright, Thomas Krieg, David Carling, Michael P. Murphy

**Affiliations:** From the ‡ MRC Mitochondrial Biology Unit, University of Cambridge, Wellcome Trust/MRC Building, Cambridge Biomedical Campus, Hills Road, Cambridge, CB2 0XY,; the § University Department of Surgery and Cambridge NIHR Biomedical Research Centre Addenbrooke's Hospital, Cambridge CB2 0QQ,; the ¶ MRC London Institute of Medical Sciences, Hammersmith Hospital, Imperial College, London, W12 0NN, and; the ‖ Department of Medicine, University of Cambridge, Addenbrooke's Hospital, Hills Road, Cambridge CB2 0QQ, United Kingdom

**Keywords:** AMP-activated kinase (AMPK), reactive oxygen species (ROS), mitochondria, hydrogen peroxide, redox signaling

## Abstract

Mitochondrial reactive oxygen species (ROS) production is a tightly regulated redox signal that transmits information from the organelle to the cell. Other mitochondrial signals, such as ATP, are sensed by enzymes, including the key metabolic sensor and regulator, AMP-activated protein kinase (AMPK). AMPK responds to the cellular ATP/AMP and ATP/ADP ratios by matching mitochondrial ATP production to demand. Previous reports proposed that AMPK activity also responds to ROS, by ROS acting on redox-sensitive cysteine residues (Cys-299/Cys-304) on the AMPK α subunit. This suggests an appealing model in which mitochondria fine-tune AMPK activity by both adenine nucleotide–dependent mechanisms and by redox signals. Here we assessed whether physiological levels of ROS directly alter AMPK activity. To this end we added exogenous hydrogen peroxide (H_2_O_2_) to cells and utilized the mitochondria-targeted redox cycler MitoParaquat to generate ROS within mitochondria without disrupting oxidative phosphorylation. Mitochondrial and cytosolic thiol oxidation was assessed by measuring peroxiredoxin dimerization and by redox-sensitive fluorescent proteins. Replacing the putative redox-active cysteine residues on AMPK α1 with alanines did not alter the response of AMPK to H_2_O_2_. In parallel with measurements of AMPK activity, we measured the cell ATP/ADP ratio. This allowed us to separate the effects on AMPK activity due to ROS production from those caused by changes in this ratio. We conclude that AMPK activity in response to redox changes is not due to direct action on AMPK itself, but is a secondary consequence of redox effects on other processes, such as mitochondrial ATP production.

## Introduction

AMP-activated protein kinase (AMPK)^2^ is a key sensor and regulator of cellular energy metabolism, which helps match mitochondrial ATP production to the energy demands of the cell (1–5). A decrease in ATP production, or an increase in its demand, elevates AMP and ADP levels relative to ATP. This results in increased binding of AMP (and ADP) to the AMPK γ subunit, which enhances AMPK activity (hereafter AMP-dependent regulation) and thus phosphorylation of a range of downstream target proteins (1–5).

**Figure 1.**
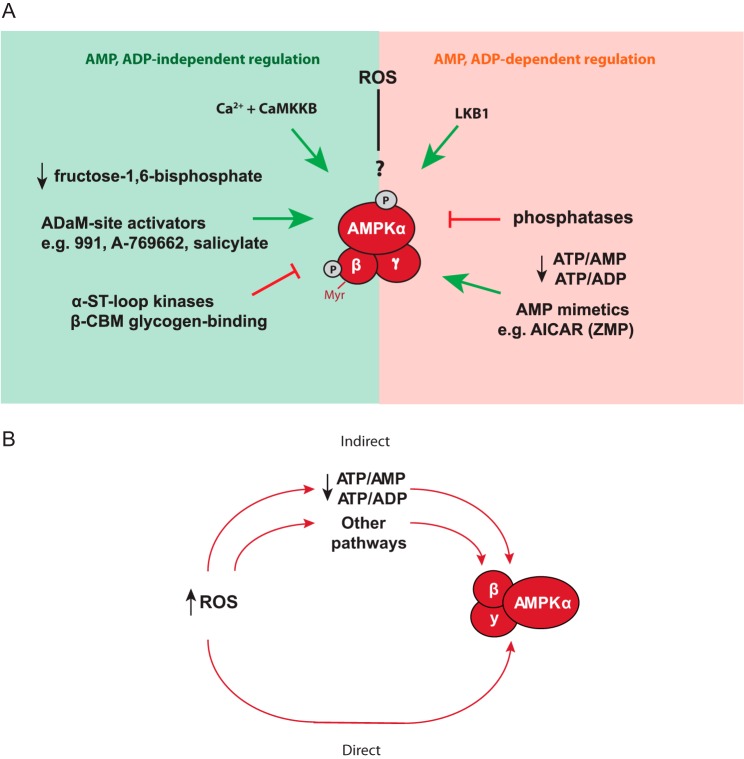
**Overview of AMPK regulation and experimental rationale.** *A,* established and hypothesized regulators of AMPK activity. *B,* summary of hypothesis showing that ROS could affect AMPK activity directly, or indirectly by altering adenine nucleotide ratios or other pathways.

AMPK can also be activated by atypical mechanisms, independently of changes in the cell's ATP/ADP or ATP/AMP ratio (hereafter AMP-independent regulation) (6–11) (Fig. 1*A*). The small molecule AMPK activators A-769662, 991, and salicylate (a metabolite of aspirin), bind at the interface of the phosphorylated β subunit carbohydrate-binding module and the N-lobe of the α subunit kinase domain, known as the Allosteric Drug and Metabolite (ADaM)-binding site (6–9). Binding of compounds at this site increases AMPK activity independently of adenine nucleotide-binding to the γ subunit by inducing a conformational change that puts AMPK into the active conformation. More recent is the finding that AMPK can also be activated in an AMP-independent manner by glucose deprivation, specifically due to a decrease in the intracellular level of fructose 1,6-bisphosphate, which promotes formation of a multienzyme complex necessary for AMPK activation (10).

It has also been proposed that reactive oxygen species (ROS) can directly regulate AMPK activity independently of changes in adenine nucleotides (12–18). Redox regulation typically occurs by the reversible oxidation of cysteine thiols, thereby altering the activity, cellular localization, or binding interactions of a protein (19–22). Mitochondria are an important site of ROS production. ROS generation by complex I through reverse electron transport (RET) is particularly attractive as a potential mitochondrial redox signal because its magnitude responds sensitively to redox status and occurs under physiological conditions (23–26). Thus, a scenario in which mitochondrial functional status and ROS production can alter AMPK activity by two parallel but independent pathways: through changes in adenine nucleotides and by redox signaling from mitochondria, has considerable appeal (Fig. 1*B*). In support of AMPK redox regulation, its activity was reported to be increased by exogenous hydrogen peroxide (H_2_O_2_), independently of changes in adenine nucleotides and this alteration required redox changes to key cysteine residues, Cys-299 and Cys-304, in the AMPK α1 catalytic subunit (13). However, other reports have challenged whether the AMPK activation by H_2_O_2_ in cells is independent of changes in the ATP/ADP or ATP/AMP ratio (17, 18) and furthermore, another laboratory has shown that exogenous H_2_O_2_ can inhibit AMPK (12). These discrepancies could arise from methodological or biological differences, such as alternative ways of measuring adenine nucleotides or different levels of ROS scavenging systems in the cells types used (12, 18). In addition, as AMPK is localized to the cytosol, it is important to assess whether changes in AMPK activity in response to ROS are associated with reversible cytosolic protein thiol oxidation, *e.g.* peroxiredoxin (Prx) dimerization (27), which would be consistent with direct ROS signaling and/or redox relay signaling (28, 29). Furthermore, mitochondrial redox signaling could also affect AMPK activity independently of either direct effects on the enzyme, or on the ATP/ADP ratio (Fig. 1*B*).

We set out to determine the possibility of AMP-independent regulation of AMPK activity in response to physiological levels of ROS generated from mitochondria in cells. To do this we altered ROS levels by addition of hydrogen peroxide. In addition, we utilized the mitochondria-targeted redox cycler MitoParaquat (MitoPQ), which can generate physiological ROS levels within mitochondria at complex I without disrupting oxidative phosphorylation (30). MitoPQ also mimics production of mitochondrial ROS at complex I by RET, a physiologically plausible mechanism of mitochondrial redox signaling (24–26, 31). To determine where protein thiol redox changes were occurring, we analyzed redox-dependent protein thiol oxidation in the mitochondria and cytosol. We further investigated the regulatory effects on AMPK activity by replacing the key redox-sensitive residues Cys-299 and Cys-304 in the AMPK α subunit (13). In parallel, we measured the cell ATP/ADP ratio to determine whether the effects on AMPK activity could be accounted for by secondary changes. These methods allowed us to separate the effects of mitochondrial ROS production on AMPK activity from those of a changing ATP/ADP ratio. We found that the effects of mitochondrial and cytosolic redox changes on AMPK activity were indirect and could be largely, but not entirely, accounted for by the effect of ROS on the ATP/ADP ratio. We conclude that ROS affect AMPK activity indirectly.

## Results

### Characterizing AMPK activity in C2C12 mouse myotubes

**Figure 2.**
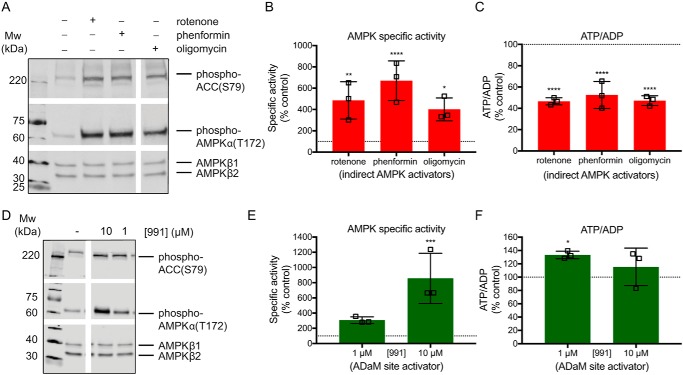
**AMPK activity markers and ATP/ADP ratios in C2C12 myotubes in response to AMP-dependent and AMP-independent activators.** *A–C,* C2C12 myotubes were treated with mitochondrial inhibitors: rotenone (2 μg/ml), phenformin (5 mm), or oligomycin (100 ng/ml) for 30 min. *A,* Western blotting of AMPKα-phospho-Thr-172, ACC-phospho-Ser-79, and AMPKβ (loading control). *B,* AMPK-specific activity was assessed by the SAMS kinase assay, measured as picomole of [γ-^32^P]ATP/μg of cell protein/min and expressed as % untreated control. *C,* ATP/ADP ratios in C2C12 myotubes were measured by a luciferase/luciferin bioluminescence assay and expressed as % untreated control. *D* and *E,* C2C12 myotubes were treated with 991 (1 or 10 μm) for 30 min. *D,* Western blotting was performed as previously described. *E,* AMPK-specific activity was measured as previously described. *F,* ATP/ADP ratios were measured as previously described. All Western blots are representative of 3 or 4 biological replicates. *Bar graphs* indicate mean ± S.D. of 3 biological replicates. Statistical analysis was performed by one-way ANOVA with Dunnett's multiple comparison post-test comparing all treatments to untreated controls; *, *p* < 0.05; **, *p* < 0.01; ***, *p* < 0.001; ****, *p* < 0.0001.

To characterize the putative regulation of AMPK activity by redox signals we used differentiated C2C12 mouse myotubes, a model of skeletal muscle fibers in which AMPK is a key regulator of mitochondrial and cellular function (11, 32, 33) (Fig. S1*A*). We first established how AMPK activity responded to the ATP/ADP ratio, which determines cellular AMP and thus accounts for AMPK regulation by adenine nucleotides (3, 4). AMPK activity was assessed by the phosphorylation of Thr-172 of AMPK's catalytic α subunit; by phosphorylation of Ser-79 on the AMPK target acetyl-CoA carboxylase (ACC); and by determining AMPK-specific activity using the SAMS kinase assay. Decreasing mitochondrial ATP production with the complex I inhibitors rotenone or phenformin or with the F_0_F_1_-ATP synthase inhibitor oligomycin all increased AMPK activity (Fig. 2, *A* and *B*). The ATP/ADP ratio in control myotubes (16.1 ± 2.1) (Fig. S1*B*) decreased on inhibition of oxidative phosphorylation to between 7.4 ± 1.1 (rotenone) and 8.3 ± 2.1 (phenformin), ∼46 and ∼52% of control cells, respectively (Fig. 2*C*). Thus, the cell ATP/ADP ratio correlates inversely with AMPK activity.

If redox signals activate AMPK independently of adenine nucleotides they will mimic pharmacological AMPK activators such as 991, A-769662, and salicylate, which bind directly to the regulatory ADaM-binding site (6–8). Treatment with 991 increased AMPK activity dose-dependently (Fig. 2, *D* and *E*) without decreasing the ATP/ADP ratio (Fig. 2*F*).

**Figure 3.**
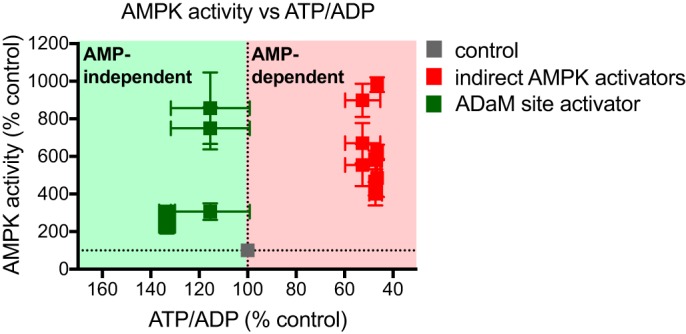
**Reference plot showing AMP-dependent (*red background*) and AMP-independent (*green background*) AMPK activation in C2C12 myotubes.** Data points are measures of AMPKα-phospho-Thr-172 (Fig. S1*H*), ACC-phospho-Ser-79 (Fig. S1*I*), and AMPK-specific activity (Fig. S1*J*) plotted against corresponding cell ATP/ADP ratios. Values are % untreated control and presented as mean ± S.E. of ≥3 biological replicates.

Combining the data where AMPK was activated indirectly (Fig. 2, *A–C*) or with the selective activator 991 (Fig. 2, *D–F*) showed a clear divergence in the relationship between the ATP/ADP ratio and AMPK activity for all three measurements of activity individually (Fig. S1, *B–G*). In Fig. S1, *H* and *I* changes in AMPK activation by alterations in the ATP/ADP ratio (AMP-dependent activation) are shown upon the red background, whereas AMP-independent activation (green background) induces AMPK activation without decreasing ATP/ADP ratios. In fact AMP-independent activation occurred with a slight increase in the ATP/ADP ratio. Combining all these data shows clearly the different trajectories of dependence of AMPK activity on ATP/ADP ratio for these two modes of regulation (Fig. 3). Next, we set out to use this approach to assess the mechanism by which redox signals affect AMPK activity.

### Exogenous H_2_O_2_ leads to dose-dependent, reversible protein thiol oxidation in the cytosol and mitochondrial matrix of myotubes

**Figure 4.**
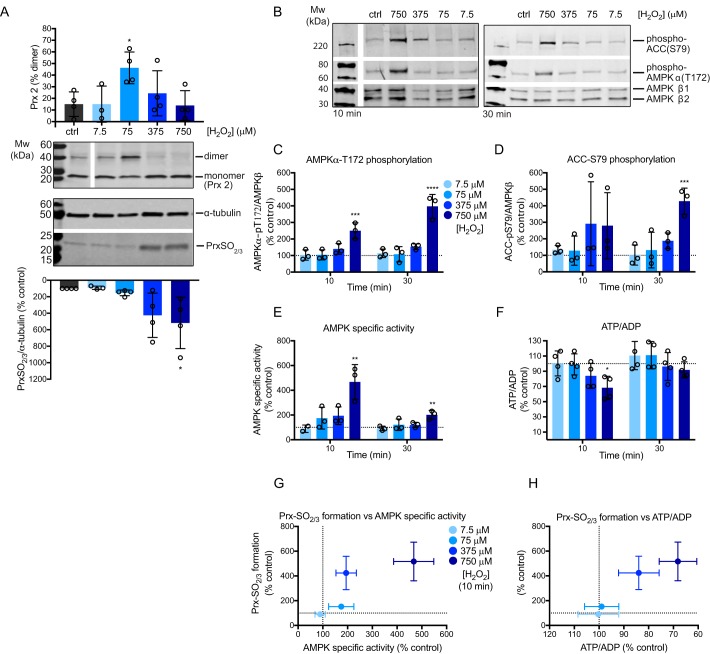
**Effects of H_2_O_2_ boluses on subcellular redox state, AMPK activity, and ATP/ADP ratios in C2C12 myotubes.** *A–H,* C2C12 myotubes were treated with serially diluted boluses of H_2_O_2_ (7.5, 75, 375, or 750 μm) in serum-free media for 10 or 30 min. *A,* Prx 2 dimerization and Prx-SO_2/3_ formation were assessed by Western blotting. *B–D,* Western blots and quantification of AMPKα-phospho-Thr-172 and ACC-phospho-Ser-79 levels in H_2_O_2_-treated cells. Graphed values are mean ± S.D. of 3 biological replicates. Results at each time point were assessed by one-way ANOVA with Dunnett's multiple comparison post-test comparing all H_2_O_2_ treatments to untreated controls; *, *p* < 0.05; **, *p* < 0.01; ***, *p* < 0.001; ****, *p* < 0.0001. *E,* AMPK-specific activity was measured as previously described. Graphed data are mean ± S.D. of 3 or mean ± range of 2 biological replicates, expressed as % untreated control. Statistical analysis was performed as described above. *F*, ATP/ADP ratios were measured as previously described. Graphed data are mean ± S.D. of 4 biological replicates, expressed as % untreated control. Statistical analysis was performed as described above. *G* and *H,* graphed data shows the relationship between Prx-SO_2/3_ formation (Fig. 4*A*) and (*E*) AMPK-specific activity or (*F*) ATP/ADP ratios in response to H_2_O_2_ in cells. Values are mean ± S.E. of ≥3 or mean ± range of 2 biological replicates, expressed as % untreated control.

We first assessed the effects of exogenous H_2_O_2_ on thiol redox status in C2C12 myotubes. Myotubes were treated with a range of H_2_O_2_ concentrations administered as single boluses. Cytosolic Prx 2 dimerization, measured 10 min after treatment, peaked upon treatment with the 75 μm H_2_O_2_ bolus (Fig. 4*A*). Extended oxidation induces thiol modifications (sulfinic acid (–SO_2_H) and sulfonic acid (–SO_3_H)), which block the formation of Prx dimers (36), hence the decrease in Prx 2 dimerization and increase in Prx-SO_2/3_ formation (Fig. 4*A*) observed in response to higher levels of H_2_O_2_. Exogenous H_2_O_2_ (≥75 μm) also oxidized the mitochondrial matrix Prx 3 pool (Fig. S2*A*). Dimers of Prx 3 were visible as double bands, likely due to the different electrophoretic mobility of dimers linked by one or two disulfide bridges (37–39).

We conclude that within the range of H_2_O_2_ concentrations tested, a period of reversible thiol oxidation occurred in both the cytosol and mitochondrial matrix, which is an environment conducive to thiol redox signaling (28, 29). The rapidly changing Prx redox states in response to single additions of H_2_O_2_ indicated rapid H_2_O_2_ metabolism. At higher H_2_O_2_ concentrations, thiol hyperoxidation occurred (Prx-SO_2/3_ formation), which is evidence of oxidative stress. We thus used this Prx redox mapping to assess the response of AMPK to protein thiol redox changes by H_2_O_2_.

### Exogenous H_2_O_2_ activates AMPK indirectly in myotubes by decreasing the ATP/ADP ratio

Addition of exogenous H_2_O_2_ that led to protein thiol oxidation in the cytosol (Fig. 4*A*) induced a dose-dependent increase in AMPK activity measured 10 min after addition of H_2_O_2_ (Fig. 4, *B–E*). H_2_O_2_ also had a dose-dependent effect on cellular ATP/ADP ratios at 10 min (Fig. 4*F*).

The dose-dependent increases in AMPK-specific activity at 10 min did not correlate with reversible Prx 2 dimerization (Fig. S2*B*), but did correlate with formation of the thiol hyperoxidation marker Prx-SO_2/3_ (Fig. 4*G*), implicating oxidative damage rather than redox signaling in the mechanism of AMPK activation. Furthermore, decreased ATP/ADP ratios at 10 min also correlated with increasing Prx-SO_2/3_ formation (Fig. 4*H*). Although H_2_O_2_ boluses lower than 750 μm were sufficient to oxidize the cytosol without affecting the cell ATP/ADP ratio, AMPK activity did not change significantly under these conditions, indicating that AMPK activity only responds to the ATP/ADP ratio and not directly to H_2_O_2_.

Of further interest, we note that 30 min after H_2_O_2_ treatment, cell ATP/ADP ratios approached recovery (Fig. 4*F*) and accordingly AMPK-specific activity decreased from that measured at 10 min (Fig. 4*E*). However, AMPK and ACC phosphorylation was sustained at 30 min (Fig. 4, *B–D*) as was Prx-SO_2/3_ formation (Fig. S2*C*).

**Figure 5.**
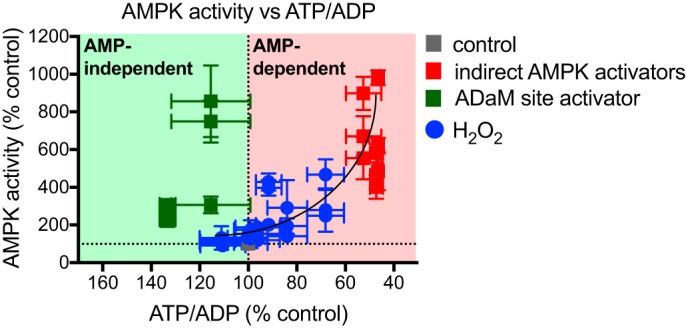
**Reference plot showing AMP-dependent and -independent AMPK activation in C2C12 myotubes.** H_2_O_2_ data points are measures of AMPKα-Thr-172 phosphorylation (Fig. S2*D*), ACC-Ser-79 phosphorylation (Fig. S2*E*), and AMPK-specific activity (Fig. S2*E*) measured at 10 and 30 min, plotted against corresponding cell ATP/ADP ratios. Values are % untreated control and presented as mean ± S.E. of ≥3 biological replicates.

All ways of measuring the changes in AMPK activity showed that addition of exogenous H_2_O_2_ increased AMPK activity (Fig. 4, *B–E*). Graphing these data confirmed that increased AMPK activity occurred only when ATP/ADP ratios were decreased (Fig. S2, *D–F*). Most significantly, the effect of exogenous H_2_O_2_ on AMPK activity was described by the same relationship as when the ATP/ADP ratio was decreased by the use of mitochondrial inhibitors (Fig. 5). We conclude that AMPK activation in C2C12 myotubes by added H_2_O_2_ occurs indirectly as a consequence of altering the cell's ATP/ADP ratio.

### Selective generation of ROS within mitochondria does not induces AMPK activation

**Figure 6.**
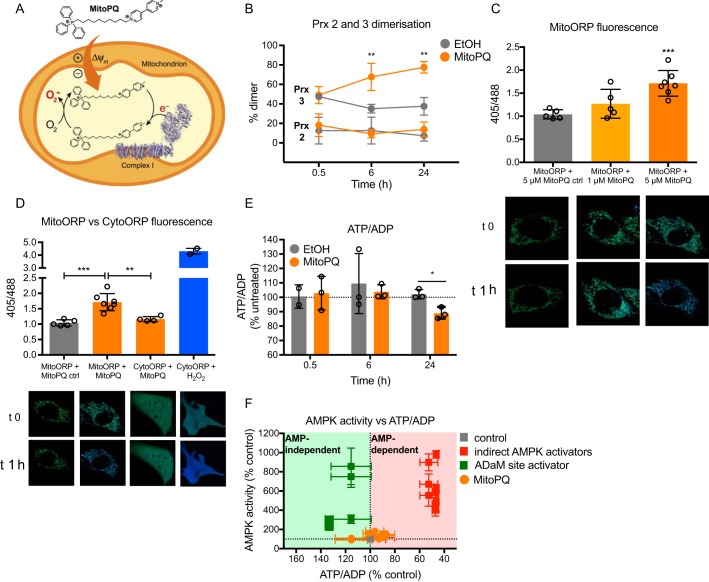
**Effects of MitoPQ on subcellular redox state, AMPK activity, and ATP/ADP ratios in C2C12 myotubes.** *A,* MitoPQ mechanism of action. *B,* C2C12 myotubes were treated with 5 μm MitoPQ or vehicle (EtOH) for 0.5, 6, or 24 h. Prx 2 and 3 dimerization was measured from Western blotting target bands (Fig. S3, *A* and *B*). Graphed data show mean ± S.D. of ≥3 biological replicates. Results at each time point were analyzed by unpaired, one-tailed *t* tests compared with EtOH controls; **, *p* < 0.01. *C* and *D,* C2C12 myoblast cells were transfected with redox-sensitive roGFP coupled to ORP1, targeted to the mitochondria (*MitoORP*) or cytosol (*CytoORP*) and treated with MitoPQ (1 or 5 μm), MitoPQ control (5 μm), or H_2_O_2_ (50 μm). roGFP-ORP1 alters excitation from 488 to 405 when oxidized by H_2_O_2_ (shown here as increasing levels of *blue*). *C,* treatment with MitoPQ induced dose-dependent oxidation of MitoORP, peaking at 5 μm. Treatment with the MitoPQ control did not oxidize MitoORP (baseline value of 1). Graphed data are mean ± S.D. of ≥5. Statistical analysis was performed by one-way ANOVA with Dunnett's multiple comparison post-test comparing all treatments to MitoPQ control; ***, *p* < 0.001. *D,* MitoPQ specifically oxidized MitoORP, with no changes observed in CytoORP. CytoORP was oxidized by addition of a single H_2_O_2_ bolus to cell media. Graphed data are mean ± S.D. of ≥4 except the H_2_O_2_ treatment, which is mean ± range of 2. Statistical analysis was performed by one-way ANOVA with Dunnett's multiple comparison post-test comparing all treatments; **, *p* < 0.01; ***, *p* < 0.001. *E,* ATP/ADP ratios in cells treated with 5 μm or EtOH control. Data are presented as mean ± S.D. of 3 biological replicates. Results at each time point were analyzed by unpaired, one-tailed *t* tests compared with EtOH controls; *, *p* < 0.05. *F,* reference plot showing AMP-dependent and -independent AMPK activation in C2C12 myotubes. MitoPQ data points are measures of AMPKα-Thr-172 phosphorylation (Fig. S3*D*) and ACC-Ser-79 phosphorylation (Fig. S3*E*) (at 6 and 24 h) plotted against corresponding cell ATP/ADP ratios. Values are % untreated control or % EtOH control (MitoPQ) and presented as mean ± S.E. of ≥3 biological replicates.

The effect of exogenous H_2_O_2_ on AMPK activity in C2C12 myotubes occurred indirectly, via changes in the ATP/ADP ratio (Fig. 5). To further analyze any effects of mitochondrial redox signals on AMPK activity, which will come from mitochondria within the cell rather than from outside the cell, we next assessed the effect of mitochondria-generated ROS on AMPK activity. Mitochondrial ROS signaling is likely to arise from enhanced superoxide generation and subsequent H_2_O_2_ production within the organelle that leads to the propagation of a signal from the mitochondria to the rest of the cell (24–26, 31). To mimic this scenario, we assessed the effects on AMPK activity of the mitochondria-targeted redox cycler, MitoPQ. MitoPQ generates superoxide selectively and continuously within mitochondria at complex I, which is then rapidly dismutated to H_2_O_2_ (30) (Fig. 6*A*). To confirm that MitoPQ generated ROS in the mitochondrial matrix and to assess potential flux of H_2_O_2_ to the cytosol, we measured the extent of redox-dependent dimerization of Prx 3 (matrix isoform) and Prx 2 (cytosolic isoform) in response to MitoPQ (Fig. 6*B* and Fig. S3, *A* and *B*). MitoPQ led to a dose- and time-dependent increase in Prx 3 dimerization from 6 to 24 h, but had no effect on Prx 2 dimerization (Fig. 6*B*), consistent with persistent ROS production in the matrix. These data indicated that MitoPQ generated ROS locally in the mitochondria, but that these ROS did not diffuse to the cytosol. Redox compartmentalization was confirmed using cytosol-specific (CytoORP) and mitochondria-specific (MitoORP) fluorescent protein probes for H_2_O_2_ (34, 35) (Fig. 6, *C* and *D*). This analysis showed enhanced H_2_O_2_-dependent fluorescence in the mitochondria in response to MitoPQ (Fig. 6*C*), but not in the cytosol (Fig. 6*D*).

There was negligible alteration to AMPK activity in response to short-term incubation with MitoPQ (Fig. S3, *C–E*). However, there were suggestions of an increase in AMPK activity upon sustained incubation with MitoPQ (Fig. S3, *C–E*). This most likely reflects the decrease in the ATP/ADP ratio upon long-term incubation with MitoPQ (Fig. 6*E*). However, the effects of MitoPQ on AMPK activity were negligible compared with either direct or indirect AMPK activators (Fig. 6*F*). That MitoPQ could significantly alter the mitochondrial thiol redox state without considerably affecting AMPK activity suggests that mitochondrial redox signaling is unlikely to regulate AMPK, either directly or indirectly.

### Mutation of the putative redox active cysteines in AMPK does not affect its activation by ROS

**Figure 7.**
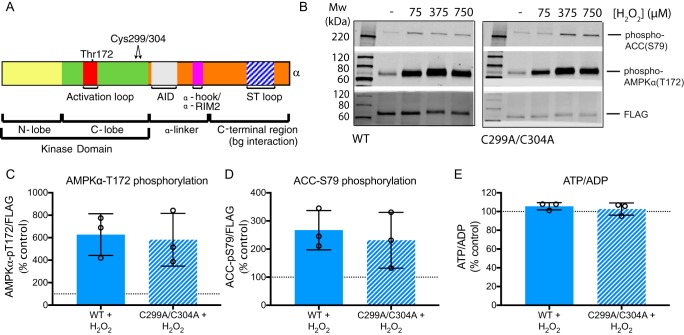
**Effects of H_2_O_2_ boluses on WT AMPK α1/β1/γ1 or AMPK α1(C299A/C304A)/β1/γ1 activity and ATP/ADP ratios in HEK 293T cells.** *A,* location of Cys-299 and Cys-304 in the AMPK α subunit. *AID*, autoinhibitory domain; *ST loop*, Ser/Thr-rich loop. *B–E,* AMPK KO HEK 293T cells overexpressing WT AMPK α1/β1/γ1 or AMPK α1(C299A/C304A)/β1/γ1 were treated with serially diluted boluses of H_2_O_2_ (75, 375, or 750 μm) in serum-free media for 10 min. *B–D,* Western blots and quantification of AMPKα-phospho-Thr-172 and ACC-phospho-Ser-79 levels in H_2_O_2_ (75 μm)-treated cells. *E,* ATP/ADP ratios in cells treated with 75 μm H_2_O_2_. Graphed values are mean ± S.D. of 3 biological replicates.

Previous studies proposed direct redox regulation of the key cysteine residues Cys-299 and Cys-304 in the α catalytic subunit of AMPK (13). To assess whether these residues could facilitate a direct effect of ROS on AMPK activation we expanded our study to an AMPK KO HEK 293T cell line and compared WT AMPK α1/β1/γ1 *versus* mutant AMPK α1(C299A/C304A)/β1/γ1 (Fig. 7*A* and Fig. S4*A*). In WT HEK 293T cells addition of exogenous H_2_O_2_ increased AMPK activity (Fig. 7, *B–D,* and Fig. S4, *B* and *C*). Importantly, the H_2_O_2_ led to identical activation of mutant AMPK α1(C299A/C304A)/β1/γ1 (Fig. 7, *B–D,* and Fig. S4, *B* and *C*). Therefore, AMPK activation by H_2_O_2_ is independent of any redox changes on the putative regulatory cysteine residues on the AMPK α subunit. The lowest concentration of H_2_O_2_ used had no effect on the ATP/ADP ratio in the HEK 293T cells (Fig. 7*E*), even though this concentration of H_2_O_2_ lead to activation of AMPK (Fig. 7, *B–D*). Higher concentrations of H_2_O_2_ affected both the ATP/ADP ratio and AMPK activity (Fig. S4, *B–D*). Therefore, these experiments show clearly that these cysteine residues on AMPK are not involved in regulating its activity. However, in contrast to C2C12 myotubes, in HEK 293T cells exogenous H_2_O_2_ can increase AMPK activity by an indirect mechanism that is distinct from changes in the ATP/ADP ratio.

### Conclusions

The appealing possibility that redox signaling from mitochondria can modulate the activity of the key energy regulator, AMPK, independently of the effects of adenine nucleotides offers possibilities of new modes of regulation of mitochondrial function. Thus, reports of the direct redox regulation of AMPK attracted considerable attention (13). However, other labs have not observed redox regulation of AMPK in response to exogenous oxidants (18), or have instead found inhibition (12). Therefore, here we set out to assess the possibility of redox regulation of AMPK in differentiated C2C12 mouse skeletal myotubes. To do this we used exogenous H_2_O_2_, or the mitochondria-targeted redox cycler MitoPQ. The rationale for using MitoPQ was that this closely mimics mitochondrial redox signaling by RET at complex I, and is thus a good model for physiological redox signaling. However, even though MitoPQ led to the selective oxidation of protein thiols within the mitochondria, suggestive of redox signaling, this did not considerably impact AMPK activity in the cytosol. Furthermore, whereas we found that exogenous H_2_O_2_ activated AMPK, this activation was not by direct redox signaling to AMPK, but was secondary to changes in cellular ATP/ADP. This was extended to HEK 293T cells where AMPK activity was also altered by exogenous H_2_O_2_. This change in AMPK activity was unaffected by replacing the putative redox-sensitive cysteine residues by redox-insensitive alanine residues. In contrast to C2C12 myotubes, in HEK 293T cells exogenous H_2_O_2_ activated AMPK independently of the ATP/ADP ratio, suggesting a further indirect response of AMPK to ROS exposure. Possible mechanisms of this response, such as changes in the level of fructose 1,6-bisphosphate (10), will be explored in future work.

Our results complement and expand previous studies into the role of ROS in AMPK activity (17, 18). In addition, some interesting new insights into AMPK regulation emerge. We observed that AMPK activity in C2C12 myotubes was increased at 10 min after addition of H_2_O_2_, which correlated with a decreased cell ATP/ADP ratio. The specific activity of AMPK later decreased at 30 min as ATP/ADP ratios recovered. As Prx-SO_2/3_ formation was increased at 30 min compared with 10 min, one possibility is that the increased Prx hyperoxidation served as a protection mechanism. A previous study has shown that under conditions of oxidative stress dimerized Prxs can compete with other cellular proteins for cysteine thiol reduction catalyzed by thioredoxins (40). Thus, Prx hyperoxidation may promote cell survival, and allow ATP/ADP ratio recovery, under conditions of oxidative stress. In agreement with a previous study (18), our data suggest that H_2_O_2_ may inhibit AMPK and ACC dephosphorylation, as AMPK and ACC phosphorylation were maintained and increased at 30 min after H_2_O_2_ addition, even as cell ATP/ADP ratios recovered.

Our work also provides insights into the redox regulation of cellular processes. Monitoring the redox states of cytosolic and mitochondrial Prx isoforms enabled us to assess the effects of redox signals on different cell compartments simultaneously and also monitor ROS flux between cell compartments. Extracellular H_2_O_2_ was capable of oxidizing mitochondrial matrix Prx 3, but only at high concentrations (≥75 μm). MitoPQ generated superoxide within the mitochondrial matrix, inducing significant Prx 3 dimerization, but there was no increase in cytosolic Prx 2 dimerization. Together these data suggest that whereas H_2_O_2_ can cross the mitochondrial inner membrane, it will more usually be degraded in the matrix by the action of Prx 3.

In conclusion, we found that the effects of redox changes on AMPK activity were indirect and could be largely, but not entirely, accounted for by the effect of ROS on the ATP/ADP ratio. We conclude that ROS affect AMPK activity indirectly.

## Experimental procedures

### Antibodies

All commercial antibodies were purchased from Cell Signaling Technology, unless otherwise stated. Primary antibodies were rabbit ACC1/2-phospho-Ser79 (number 3661); rabbit ACC1/2 (number 3662); rabbit AMPKα1/2-phospho-Thr-172 mAb 40H9 (number 2535); rabbit AMPKα1/2 (number 2532); rabbit-AMPKβ1/2 mAb 57C12 (number 4150); mouse α-tubulin clone B-5–1-2 (Sigma: number T5168); mouse FLAG M2 (Sigma, number F1804); mouse Myc (Merck, number 05724); mouse Prx 2 (Abcam: number ab50862); rabbit-Prx 3 (Abcam: number ab73349); rabbit Prx-SO_2/3_ (Cambridge Research Biochemicals: number crb2005004e); rabbit myogenin (Abcam, number ab124800); and rabbit anti-pan-AMPKβ antibody (42). Secondary antibodies were IRDye® 800 goat anti-rabbit IgG F(c) (Rockland Antibodies and Assays: number 611-132-003) and IRDye® 680RD goat anti-mouse IgG (H+L) (LI-COR Biosciences: number 926–68070).

### Plasmids

cDNA encoding WT human AMPKα1, or harboring mutation of cysteine residues 299 and 304 to alanine, were cloned into pLPC, and engineered to encode an N-terminal FLAG epitope tag. cDNAs encoding rat AMPKβ1 and rat AMPKγ1 (harboring an N-terminal Myc epitope tag) were cloned into pCDNA3. All constructs were confirmed by DNA sequencing.

### Cell culture

HEK 293T AMPK knockout (KO) cells were generated by using CRISPR/Cas9 to remove expression of both AMPKβ1 and -β2, as previously described (41). Transient transfections were performed using Lipofectamine® 2000 (according to the manufacturer's instructions), 24 h prior to cell treatments. C2C12 mouse myoblast cells and HEK 293T cells were cultured in standard Dulbecco's modified Eagle's medium/GlutaMAX medium (glucose (4.5 g/liter), sodium pyruvate (100 mg/ml)) supplemented with 10% (v/v) fetal bovine serum (FBS), penicillin (100 units/ml), and streptomycin (100 μg/ml). Cells were incubated in a humidified atmosphere (5% CO_2_, 95% air) at 37 °C. C2C12s were differentiated to myotubes by serum depletion to 1% (v/v) FBS for 7 days, with media changes every 2 days. Treatments were performed on day 7 or 8 of serum depletion. H_2_O_2_ treatments were performed in serum-free medium. After treatments, cells were rapidly washed in ice-cold PBS before rapid lysis on an ice-cold aluminum block. For AMPK assays, cells were lysed in 250 μl of ice-cold Hepes lysis buffer: Hepes (50 mm, pH (7.4)), EDTA (1 mm), glycerol (10% (v/v)), sodium fluoride (50 mm), sodium pyrophosphate (5 mm), Triton X-100 (1% (v/v)). This was supplemented just before use with dithiothreitol (DTT) (1 mm) and protease inhibitors: soybean trypsin-chymotrypsin inhibitor (4 μg/ml), phenylmethanesulfonyl fluoride (0.1 mm), and 1 mm benzamidine (1 mm). Cells for Prx oxidation assays were lysed in 250 μl of RIPA buffer: Tris (50 mm, pH 8.0, at 4 °C), sodium chloride (150 mm), Triton X-100 (1.0% (v/v)), sodium deoxycholate (0.5% (w/v)) and SDS (0.1% (w/v)). This was supplemented just before use with protease inhibitors and methyl methanethiosulfonate (80 mm). Prior to lysis, cells were incubated with methyl methanethiosulfonate (80 mm) for 5 min at room temperature. Cells for ATP/ADP assays were lysed in 500 μl of ice-cold perchloric acid extractant: HClO_4_ (3% (v/v)), Na_2_EDTA (2 mm), and Triton X-100 (0.5% (v/v)). Lysed cell supernatants were stored on ice for immediate use or aliquoted, snap-frozen on dry ice, and stored at −20 or −80 °C (long-term).

### SDS-PAGE

Cell lysate was heated in Laemmli sample buffer with freshly added DTT (25 mm) for 5 min at 95 °C (DTT was excluded for Prx Western blots). Samples (∼25 μg of protein) were loaded on Mini-PROTEAN® TGX^TM^ Precast 4–20% gradient gels (Bio-Rad). Proteins were electrophoresed at 100 volts in the presence of Tris (25 mm, pH (8.3)), glycine (192 mm), and SDS (0.1% (w/v)).

### Western blotting

Electrophoresed proteins were transferred to Immobilon®-FL PVDF membranes by wet transfer performed at 100 volts for 1 h at 4 °C in the presence of Tris (25 mm), glycine (192 mm), and methanol (20% (v/v), pH 8.4) at 4 °C. Where appropriate, membranes were sectioned according to molecular weight before incubation with primary antibodies, overnight at 4 °C. Signal intensities of target bands were measured as fluorescence emission at 800 or 680 nm with the Odyssey CLx IR Imaging System and quantified with LI-COR Biosciences Image Studio Lite software. In C2C12 lysates, phospho-AMPKα1/2(Thr-172) and phospho-ACC1/2(Ser-79) signal intensities (SI) were normalized to AMPKβ2. In HEK 293T cells, overexpressed phospho-AMPKα1(Thr-172) and endogenous phospho-ACC1/2(Ser-79) SIs were normalized to overexpressed AMPKα1-FLAG. Prx dimerization was calculated as % dimer (SI dimer/(SI dimer + SI monomer) × 100). Prx-SO_2/3_ formation was normalized to α-tubulin.

### AMPK SAMS kinase assay

The specific kinase activity of AMPK was determined by the radiometric SAMS peptide assay (43). Kinase assays were performed on AMPK immunoprecipitated from 100 to 200 μg of whole cell lysate using a rabbit-pan β antibody conjugated to protein-A (from *Staphylococcus aureus*)-Sepharose beads. Kinase assays were performed in duplicate and normalized to SAMS-blank controls.

### ATP/ADP assays

ATP and ADP levels were measured by luciferase/luciferin bioluminescence using an AutoLumat LB-953-Plus multi-tube luminometer (Berthold), fitted with an autoinjector, and quantified against standard curves of purified ATP and ADP. For ATP measurements, 100 μl of lysate was added to 400 μl of Tris acetate buffer (Tris (100 mm), Na_2_EDTA (2 mm), MgCl_2_ (50 mm), pH 7.75) with glacial acetic acid) in luminometer tubes. Luciferase/luciferin solution (DTT (7.5 mm), BSA (0.4 mg/ml), firefly luciferase (1.92 μg of protein/ml), and d-luciferin (120 μm) was made just before use and 100 μl was delivered to each sample tube via autoinjection. Reactions were performed at 30 °C and light emission (relative light units) was recorded 30 s post-injection. ADP was measured by first degrading endogenous ATP with 2× ATP sulfurylase solution followed by incubation at 30 °C for 30 min with agitation (500 rpm) and then heat inactivation. 200 μl of each ATP sulfurylase-treated sample was added to 400 μl of Tris acetate buffer in luminometer tubes (in duplicate). To convert endogenous ADP to ATP, 10 μl of pyruvate kinase/phospho(enoyl)pyruvate solution (type II protein kinase from rabbit muscle (5 units), phosphoenolpyruvate (100 mm)) was added to one of the duplicate tubes (and to all ADP standards) and incubated at 30 °C for 30 min prior to ATP measurement. The protein kinase/phosphoenolpyruvate-blank tubes served as blanks for quantification. Quantification was performed with Excel or GraphPad Prism 7.0. ATP and ADP values were expressed as ATP/ADP ratios.

### Microscopy

C2C12 mouse myoblast cells were seeded at a density of 20,000 cells on pre-coated glass coverslips in 6-well plates (Nunclon Delta Surface, Thermo Scientific). 24 h after plating, cells were transfected with Lipofectamine® 2000 and 1 μg of a plasmid encoding either the cytoplasmic targeted roGFP2 ORP1, or the mitochondria-targeted roGFP2 ORP1 (34) (Addgene numbers 64993 and 64992). 48 h after transfection, C2C12 cells were imaged using a Zeiss LSM microscope every 30 s for 1 h in Dulbecco's modified Eagle's medium + 10% FBS. Compounds were added immediately prior to imaging and remained present throughout. The roGFP was sequentially excited at 405 and 488 nm with emission collected at 500–550 nm for both excitation wavelengths. Images were analyzed in ImageJ where the channels were split dependent upon excitation, regions of interest were drawn around the cells, and fluorescence intensity was measured across the full hour. A ratio of 405/488 fluorescence was calculated before the initial intensity was normalized to 1, and subsequent time points were calculated as a derivative. Data are mean ± S.D.

### Statistical analysis

Data analysis was performed with GraphPad Prism 7.0 (unless otherwise stated). Data (*n* = 2) were expressed as mean ± range. Data (*n* ≥ 3) were analyzed by unpaired, one-tailed *t* tests (2 groups) or one-way analysis of variance (ANOVA) (>2 groups) unless otherwise stated. Data were expressed as mean ± S.D. (bar graphs) or mean ± S.E. (*xy* graphs). *p* values < 0.05 were considered statistically significant.

## Author contributions

E. C. H. and M. P. M. conceptualization; E. C. H. data curation; E. C. H. formal analysis; E. C. H. and M. P. M. supervision; E. C. H., A. V. G., N. N., A. R. H., G. B., T. P. B., and T. K. investigation; E. C. H., A. V. G., R. W., N. N., A. R. H., G. B., T. P. B., T. K., and D. C. methodology; E. C. H. writing-original draft; E. C. H., and M. P. M. project administration; E. C. H., D. C., and M. P. M. writing-review and editing; R. W., N. N., and D. C. resources; M. P. M. funding acquisition.

## Supplementary Material

Supporting Information

## Footnotes

2 The abbreviations used are:

AMPK: AMP-activated protein kinase
ROS: reactive oxygen species
ADaM: allosteric drug and metabolite
RET: reverse electron transport
Prx: peroxiredoxin
ACC: acetyl-CoA carboxylase
FBS: fetal bovine serum
SI: signal intensities
ANOVA: analysis of variance'
roGFP: reduction-oxidation sensitive green fluorescent protein.
