# Supplementary material for: Mitochondria-derived ROS activate AMP-activated protein kinase (AMPK) indirectly
Source: J Biol Chem. 2018 Sep 19;293(44):17208–17. doi: 10.1074/jbc.RA118.002579 (PMC6222118; doi:10.1074/jbc.RA118.002579)
Supplement: Supporting Information [file supp_293_44_17208__index.html]

Mitochondria-derived ROS activate AMP-activated protein kinase (AMPK) indirectly — ROS activate AMPK indirectly — Mitochondria-derived ROS activate AMP-activated protein kinase (AMPK) indirectly — ROS activate AMPK indirectly — Supporting Information 

# Mitochondria-derived ROS activate AMP-activated protein kinase (AMPK) indirectly

## Supporting Information

- Supporting Information (to be published online) - Supplementary Figures 1 - 4
