# Supplementary material for: Mitochondria-derived ROS activate AMP-activated protein kinase (AMPK) indirectly
Source: J Biol Chem. 2018 Sep 19;293(44):17208–17. doi: 10.1074/jbc.RA118.002579 (PMC6222118; doi:10.1074/jbc.RA118.002579)
Supplement: Supporting Information [file supp_RA118.002579_136224_2_supp_203096_pf9gvc.pdf]

Mitochondria-derived ROS activate AMP-Activated Protein Kinase (AMPK) indirectly

Elizabeth C. Hinchy[a], Anja V. Gruszczyk[a,b], Robin Willows[c], Naveenan Navaratnam[c], Andrew R. Hall[a], Georgina Bates[a], Thomas P. Bright[a], Thomas Krieg[d], David Carling[c], Michael P. Murphy[a, 1]

[a]MRC Mitochondrial Biology Unit, University of Cambridge, Wellcome Trust/MRC Building, Cambridge Biomedical Campus Hill Road, Cambridge, CB2 0X, UK
[b] University Department of Surgery and Cambridge NIHR Biomedical Research Centre, Addenbrooke's Hospital, Cambridge CB2 0QQ, UK
[c] MRC London Institute of Medical Sciences, Hammersmith Hospital, Imperial College, London, W12 0NN, UK
[d] Department of Medicine, University of Cambridge, Addenbrooke's Hospital, Hills Road, Cambridge CB2 0QQ, UK

Supporting Information

Supplementary Figures S1-S4

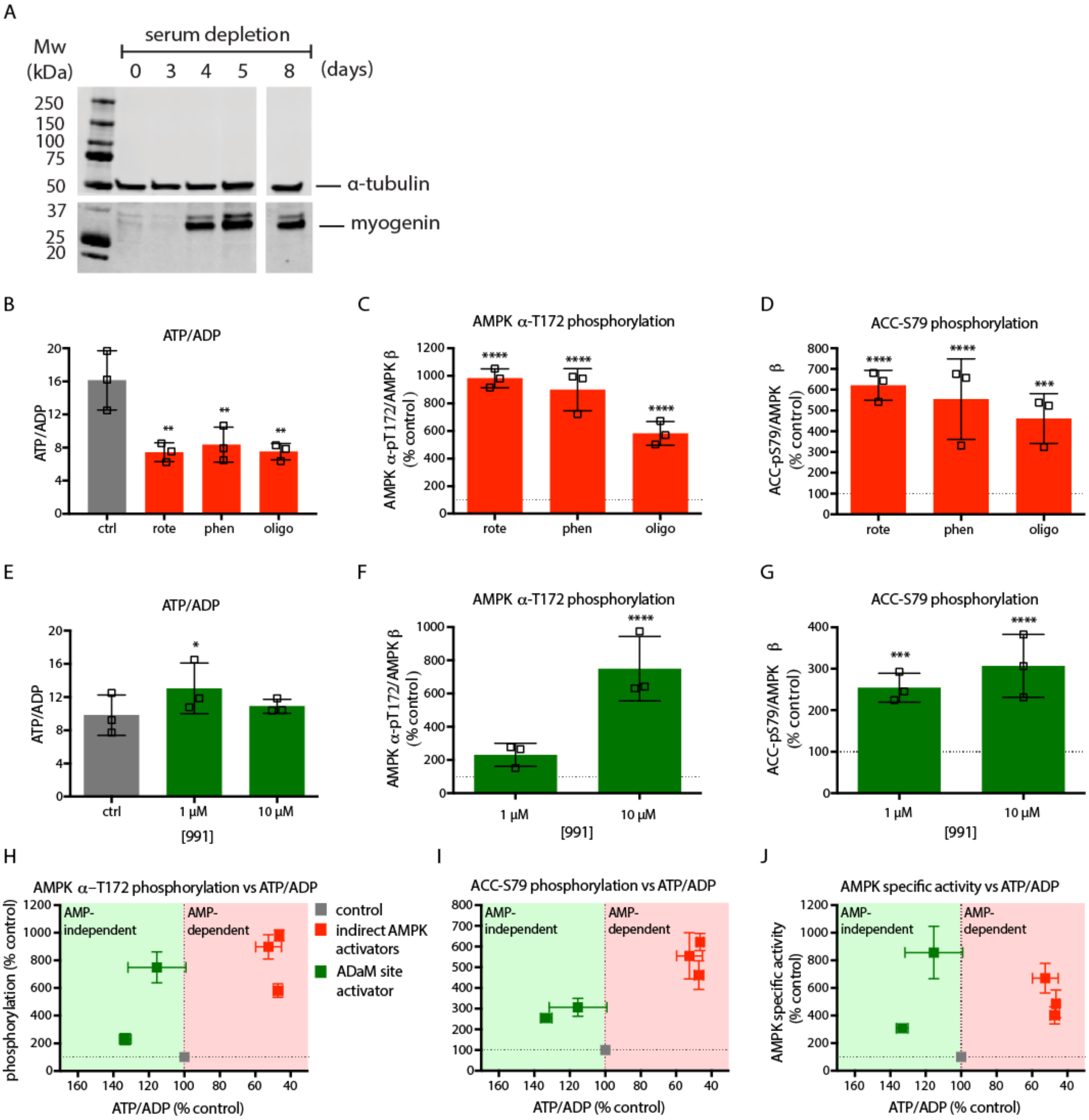

FIGURE S1. ATP/ADP ratios, AMPK activity and reference plots showing their relationships in C2C12 myotubes in response to AMP-dependent and -independent activators. (A) C2C12 differentiation confirmed by expression of myogenin. (B - D) Cells were treated with mitochondrial inhibitors: rotenone (2 μg/ml), phenformin (5 mM) or oligomycin (100 ng/ml) for 30 min. ATP/ADP ratios were measured by bioluminescence. (C) AMPKα-phosphoT172 and (D) ACC-phosphoS79 quantification from blots (Fig. 2A). Graphs indicate mean ± SD of 3 biological replicates. Statistical analysis was performed by one-way ANOVA with Dunnett's Multiple Comparison post-test comparing all treatments to untreated controls; **P < 0.01, ***P < 0.001, ****P < 0.0001. (E - G) Cells were treated with 991 (1, 10 μM) for 30 min. (E) ATP/ADP ratios were measured as above. (F) AMPKα-phosphoT172 and (G) ACC-phosphoS79 quantification from blots (Fig. 2D). Graphs indicate mean ± SD of 3 biological replicates. Statistical analysis was performed by one-way ANOVA with Dunnett's Multiple Comparison post-test comparing all treatments to untreated controls; **P < 0.01, ***P < 0.001, ****P < 0.0001. (H - I) Reference plots showing AMP-dependent and -independent AMPK activation in C2C12 myotubes. Data points are measures of (H) AMPKα-T172 phosphorylation, (I) ACC-S79 phosphorylation and (J) AMPK specific activity plotted against corresponding cell ATP/ADP ratios. Values are % untreated control and presented as mean ± SEM of 3 biological replicates.

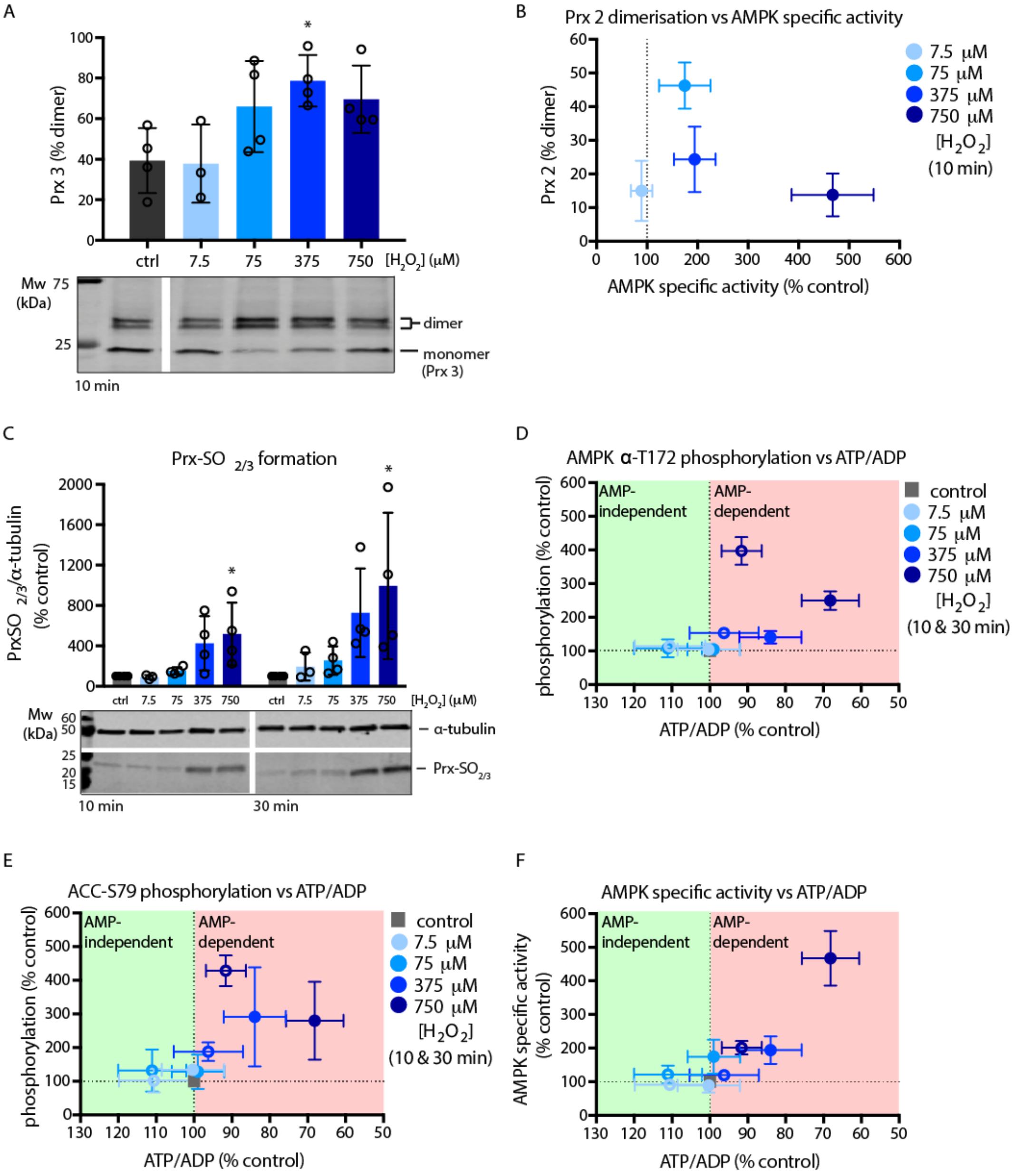

FIGURE S2. Effects of H_2O_2 boluses on subcellular redox state and reference plots showing the relationship between AMPK activity markers and ATP/ADP ratios in C2C12 myotubes in response to H_2O_2. (A – F) C2C12 myotubes were treated with serially diluted boluses of H_2O_2 (7.5, 75, 375 or 750 μM) in serum-free media for 10 or 30 min. (A) Prx 3 dimerisation was assessed by Western blotting. (B) Graphed data shows the relationship between Prx 2 dimerisation and AMPK specific activity in response to H_2O_2. Values are mean ± SEM of ≥ 3 or mean ± range of 2 biological replicates. (C) Prx-$SO_{2/3}$ formation at 10 or 30 min post H_2O_2 addition was assessed by Western blotting. (D) AMPKα-T172 phosphorylation, (E) ACC-S79 phosphorylation and (F) AMPK specific activity, measured at 10 min (opaque) or 30 min (outlined), plotted against corresponding cell ATP/ADP ratios. Values are % untreated control and expressed as mean ± SEM of ≥ 3 biological replicates.

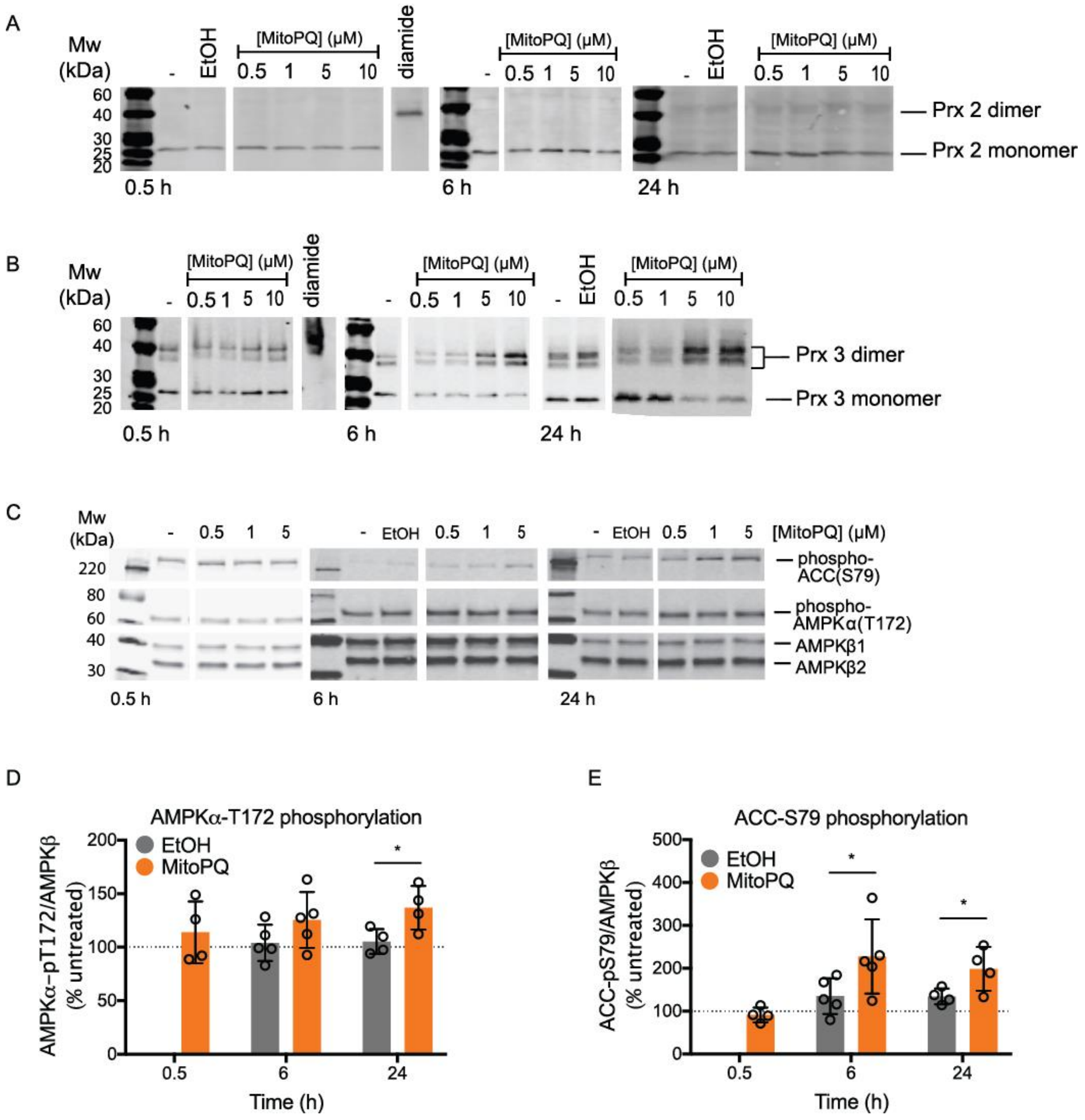

FIGURE S3. Effects of MitoPQ on subcellular redox state and AMPK activity in C2C12 myotubes in response to MitoPQ. (A & B) Western blots of Prx 2 and Prx 3 dimerisation in C2C12 myotubes treated with MitoPQ (0.5, 1, 5 or 10 μM), or EtOH vehicle control, or diamide (500 uM), for 0.5, 6 or 24 h. (C - E) Western blots and quantification of AMPKα-phosphoT172 and ACC-phosphoS79 levels in MitoPQ treated cells. Graphed values (5 μM MitoPQ versus EtOH control) were presented as mean ± SD of $n \geq 3$ biological replicates. Results at each time-point were analysed by unpaired, one-tailed t tests compared to EtOH controls; *$P < 0.05$.

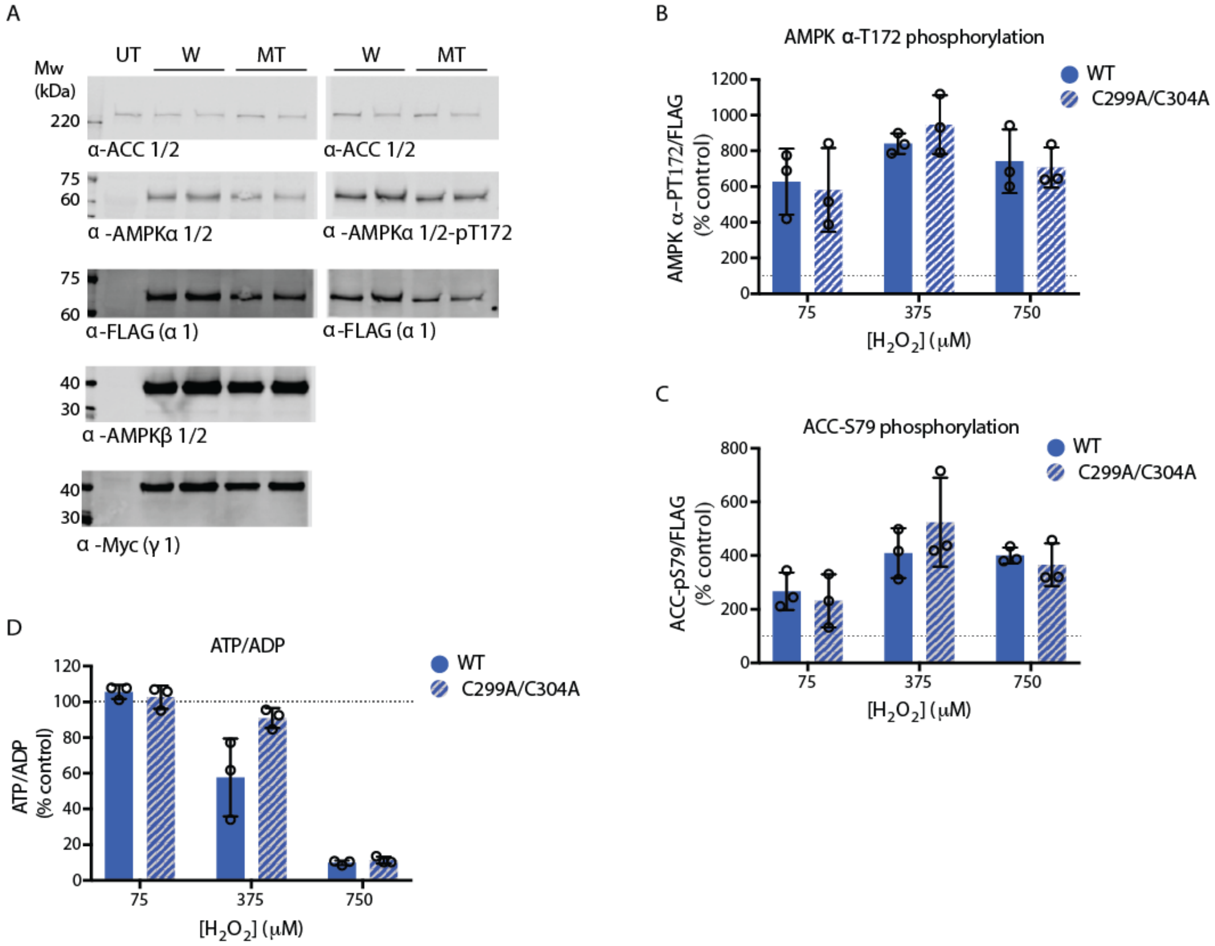

FIGURE S4. Expression of wild-type or mutant AMPK in the AMPK K/O HEK 293T cell line, and effects of H_2O_2 boluses on wild-type and mutant AMPK-expressing cells. (A - D) AMPK K/O HEK 293T cells (UT) were transiently co-transfected with AMPK α1 (WT-FLAG (1 μg DNA) (WT) or C299A/C304A-FLAG (2 μg DNA) (MT)) and AMPK β1 (1 μg DNA) and AMPK γ1-Myc (1 μg DNA) per plate, 24 h prior to treatment and/or lysis. (B – D) Cells were treated with serially diluted boluses of H_2O_2 (75, 375 or 750 μM) in serum-free media for 10 min. Graphed values are mean ± SD of 3 biological replicates. (B & C) Results were analysed by two-way ANOVA; H_2O_2 affected AMPKα-phosphoT172 and ACC-phosphoS79 levels (****$P < 0.0001$ and **$P < 0.01$, respectively) but the α1 C299A/C304A mutation did not.
